# Genomic and epidemiological perspectives on the first local sporadic cases of Mpox in China

**DOI:** 10.1080/22221751.2023.2245932

**Published:** 2023-08-21

**Authors:** Hongling Jia, Tong Sha, Shilei Zhao, Wenzhe Su, Peiwen Liu, Ruonan Zhen, Peihan Li, Lijuan Zhou, Yang Xu, Yunjing Wen, Lianjiang Chi, Biao Di, Peng Li, Hua Chen, Pengzhe Qin

**Affiliations:** a Guangzhou Center for Disease Control and Prevention, Guangzhou, People’s Republic of China; b Department of Medical Biochemistry and Molecular Biology, School of Medicine, Jinan University, Guangzhou, People’s Republic of China; c Beijing Institute of Genomics, Chinse Academy of Sciences and China National Center for Bioinformation, Beijing, People’s Republic of China; d School of Future Technology, University of Chinese Academy of Sciences, Beijing, People’s Republic of China; e Chinese PLA Center for Disease Control and Prevention, Beijing, People’s Republic of China

**Keywords:** MPXV, phylogenetic analysis, haplotype network, genomic epidemiology, covert transmission

## Abstract

From June 7th to 11th, 2023, eight cases of Mpox were identified in Guangzhou, China. This is the first report of multiple local sporadic cases after the imported case in Chongqing, China. Epidemiological investigation revealed that these cases had no history of international travel and no connections with each other. Haplotype network and phylogenetic analyses indicated that the possible origin is likely from Japan, although the direct origin may remain uncertain due to limited genomic sequences and sampling bias in GISAID. The three Guangzhou sequences have accumulated several novel mutations, suggesting the local transmission of Mpox may have been ongoing for some time. Based on the daily cases during the early stage of Mpox outbreak in four other countries, the number of possible infected cases in Guangzhou is inferred to be more than 300, suggesting that swift and efficient control measures must be implemented to mitigate the risk of a potential epidemic.

## GRAPHICAL ABSTRACT

**Figure:**
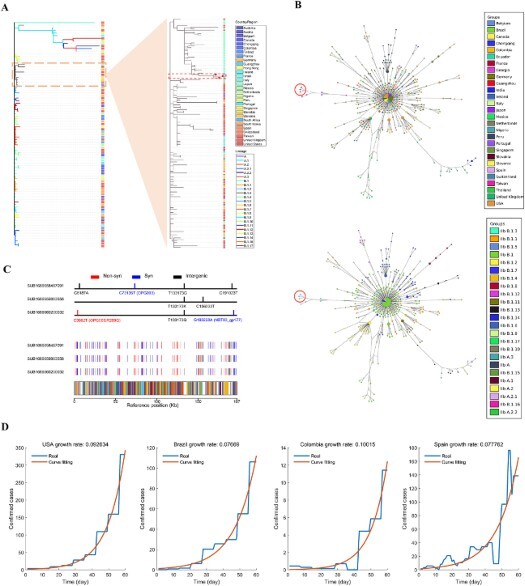

Mpox is a zoonosis caused by Mpox virus (MPXV, previously known as Monkeypox), a member of the genus *Orthopoxvirus* in the family *Chordopoxvirinae*, which was first identified in 1958. MPXV infection in humans generally manifests as fever, headache, lymphadenopathy, muscle soreness, pocks and some genitourinary symptoms, including dysuria, urgency, increased urinary frequency, haematuria, and herpetic lesions around the genitals [1]. Before 2022, MPXV infection was uncommon, especially between humans. The most common way of exposure was direct or indirect contact with Mpox-infected wild animals. In 2022, an outbreak of Mpox among men who have sex with men (MSM) has drawn the attention of the public and governments all over the world to its epidemiological characteristics and associated risk factors within this specific subpopulation. MPXV has spread over 111 countries and regions worldwide, with 87,979 reported infected cases as of June 12th, 2023 [2] (data source: Our World in Data, https://ourworldindata.org/monkeypox).

The first imported case of Mpox in mainland China was identified and reported in Chongqing on September 16th, 2022 [3]. In Guangzhou, the largest city in South China, we have identified eight Mpox cases among MSM from June 7–11 in 2023. A detailed epidemiological investigation was carried out to gather comprehensive information about their sexual behaviour in the 21 days preceding the symptom onset, exposure to known Mpox cases, international travel history, and exposure to rodents or non-human primates. Additionally, participants were assessed for previous history of sexually transmitted infections, such as HIV or syphilis.

Epidemiology investigation showed that all eight cases in this study had a history of homosexual intercourse, and five of them had more than one homosexual mate. Additionally, seven cases had other sexually transmitted diseases such as syphilis, genital herpes, condyloma acuminatum, or HIV infection (Supplement Table S1). Environmental swabs were collected in the residences of four cases, three of which were MPXV-positive. However, none of the eight individuals showed epidemiological links to each other or international travel history; furthermore, their places of residence are scattered in different areas of Guangzhou.

Whole-genome sequencing of the eight samples were conducted on the Illumina Miniseq Platform, and three full-length genomes of MPXV were successfully obtained (accession numbers: NMDCN0001DQB, NMDCN0001DQ9, and NMDCN0001DQA). Eighty-seven representative genomes covering a variety of lineage and regions were retrieved from the GISAID database (https://gisaid.org/), and aligned with the three MPXV genomes from Guangzhou and the reference genome NC063383.1 using Nextclade version 2.14.1 [4]. The phylogenetic tree was constructed using MEGA 11 with the NJ method [5]. The result showed that the three MPXV genomes from Guangzhou belong to Clade IIb, Linage B.1.3 (Figure 1(A)). To trace the possible origin, a second phylogenetic tree was further constructed for the 145 sequences belonging to the B1.1.3 lineage in GISAID, and the genomes of the three cases showed the closest relationship with a case collected from Japan (GISAID ID: EPI_ISL_17692269), indicating that the Guangzhou cases may be transmitted from some case imported from Japan (Figure 1(A)). Haplotype network was constructed using fastHaN [6]. The Guangzhou cases cluster together and are close to the samples collected from Japan (Figure 1(B)). It also hints a possible single origin given the cluster of the three sequences. However, only a proportion of Mpox cases was sequenced and deposited in GISAID; more data are needed to help refine the origin and trace the transmission chain.

**Figure 1.**
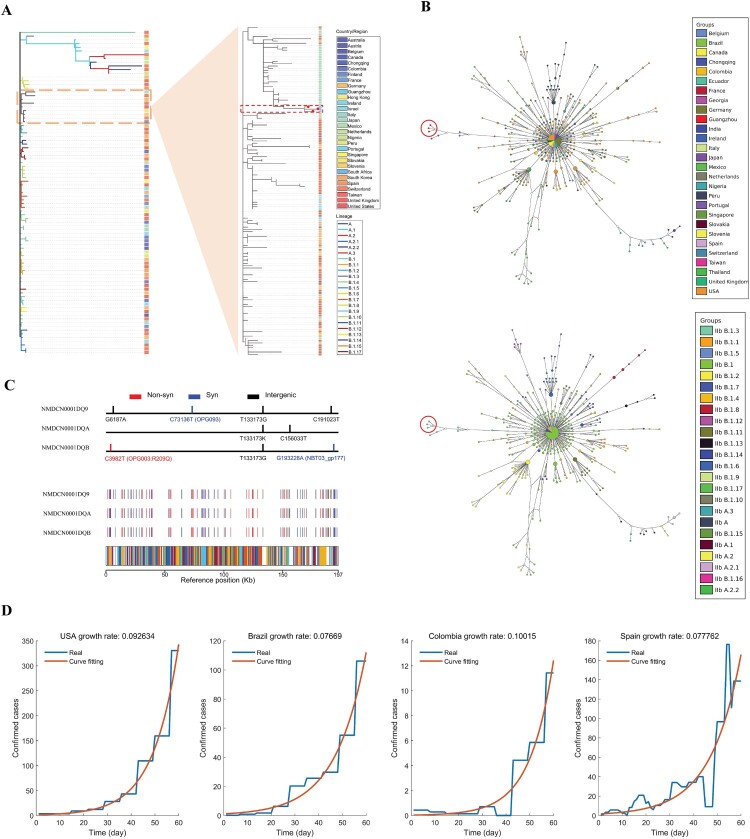
Genomic and epidemiological analysis of the three Guangzhou MPXV samples and other worldwide data. (A) The phylogenetic trees of MPXV sequences. Branches within the red box are the Guangzhou sequences; (B) The haplotype network of MPXV sequences. Nodes within the red circle are the Guangzhou sequences; (C) The polymorphism sites identified in the three Guangzhou samples; (D) The daily growth rates inferred by fitting an exponential growth to the daily confirmed cases of four representative countries.

Compared to the reference genome, the three cases have 85, 86, and 83 mutations, respectively. The non-synonymous mutation numbers are 37, 36, and 36, respectively (Supplement Table S2). Compared to the closest sequence from Japan, the three samples shared one mutation (T133173G). There are five mutations among the three sequences, one of which is a nonsynonymous mutation (C3982 T) located on the gene OPG003 (R209Q) (Figure 1(C)). The five mutations are all novel without being identified in other samples, further supporting a possible single origin of these sporadic cases. The divergence among the three Guangzhou sequences indicated that Mpox may have been locally transmitting for some time. We thus inferred the time to the most recent common ancestor (TMRCA) of the three MPXV sequences using virusMuT [7]. By assuming a point mutation rate of 13.5 per genome per year [8], the TMRCA of the three sequences were inferred to be 54.07 days before June 11th, 2023 (95% confidence interval: 12.80-99.36). If these sporadic cases were from a single imported source, then the transmission of Mpox has been in Guangzhou for around 8 weeks.

We further inferred the total number of infected cases in Guangzhou. Since there is limited information about the reproduction number and infection cycle of Mpox, we inferred the daily growth rates of Mpox by fitting an exponential growth model to the reported numbers of daily cases during their early stage (the first 60 days) in four countries (United States, Brazil, Colombia and Spain, data source: Our World in Data https://ourworldindata.org/monkeypox). The inferred daily growth rates are 0.093, 0.077, 0.100, and 0.078, respectively (Figure 1(D)). If we assume a single-origin scenario for Guangzhou sporadic cases, and take the average daily rate of 0.0868, and the TMRCA of 54.07 days (95% confidence interval: 12.80–99.36), the estimated number of locally infected cases by the sampling date is 1020 (95% confidence interval: 22–43,700). The inferred scale of infection indicates the risk of a potential outbreak in Guangzhou. However, the spread of Mpox seems much slower than COVID-19, and the susceptible population of Mpox is much smaller, the scale of the epidemic may be small, and the trend be transient.

Overall, we reported a comprehensive genomic and epidemiological investigation of eight sporadic Mpox cases in Guangzhou, China. This is the first report of multiple local sporadic cases of Mpox after the imported cases reported in Chongqing, China. Our analysis outlines the characteristic of the local sporadic cases: First, all the eight cases reported no recent oversea travel history, indicating a local transmission, while the original imported cases have not yet been found. Second, haplotype network and phylogenetic analyses indicated that the possible origin is likely from Japan, although the direct origin may be missing due to current limited genomic sequences and sampling bias in GISAID. Third, the three Guangzhou sequences have accumulated several novel mutations, suggesting the local transmission of Mpox has been going on for some time. Our quantitative analysis predicts hundreds of infection cases in the population, which could lead to local epidemic if efficient control measures are not implemented.

On May 11th, 2023, WHO announced that Mpox epidemic no longer constitutes an international public health emergency of concern [9]. However, the recent spread and hidden transmission of Mpox in Guangzhou alarmed potential Mpox epidemics in China caused by imported cases and posed prompt action to prevent it. Furthermore, the pattern of sexual transmitted infections among MSM subpopulation renders more challenges to the identification of imported and domestic infected Mpox cases, especially the collection of epidemiological information. To effectively mitigate the outbreak, it is imperative to enhance the Mpox surveillance system and improve the early detection capability of medical institutions. Additionally, public awareness and education campaigns targeting MSM communities should be strengthened to reduce the risk of sustained transmission [10,11].

## Supplementary Material

Supplemental Material

Supplemental Material

Supplemental Material
