# Supplementary material for: Genomic and epidemiological perspectives on the first local sporadic cases of Mpox in China
Source: Emerg Microbes Infect. 2023 Aug 21;12(2):2245932. doi: 10.1080/22221751.2023.2245932 (PMC10443958; doi:10.1080/22221751.2023.2245932)
Supplement: Supplemental Material [file TEMI_A_2245932_SM6495.docx]

**Methods**

**Ethics.**

This study was approved by the Ethics Committee of the Center for Disease Control and Prevention (CDC) of Guangzhou (GZCDC-ECHR-2023P0035). All participants have signed informed consent forms.

**Genome sequencing**

Rash fluids, serum samples, and throat swabs of the eight cases were collected and sent to Guangzhou Center for Disease Control and Prevention (GZCDC) for MPXV specific real-time PCR detection and MPXV whole genome sequencing (WGS). Viral DNA was extracted directly from the MPXV-positive samples with QIAamp MinElute Virus Kits (Qiagen, 57704) and the DNA libraries for Illumina Platform were constructed with QIAseq FX DNA library Kit (Qiagen, 180479). Metagenomic single-end 101 cycles (SE150) sequencing of these libraries was performed on the Illumina Miniseq platform. Three full-length genomes of MPXV were successfully obtained (~~CNMBDC~~ accession numbers: NMDCN0001DQB, NMDCN0001DQ9, and NMDCN0001DQA).

**Constructing the phylogenetic tree**

We selected 87 representative MPXV sequences covering all major lineages and regions from GISAID. Sequences were aligned and annotated against a reference genome (NC063383.1) using Nextclade version 2.14.1 [1]. The tree was built using MEGA 11 [2], and the final visualization was enhanced using iTOL version 6.7.5 [3] . A separate phylogenetic tree was constructed using 145 sequences belonging to the B.1.3 lineage with the same method.

**Constructing the haplotype network**

A subset of 507 complete MPXV sequences was randomly selected from 3040 current sequences available on GISAID. These 507 sequences and the three sequences from Guangzhou were used to build the haplotype network using fastHaN [4]. The inferred network was further visualized using tcsBU [5].

**Estimating the time to the most recent common ancestor (TMRCA)**

We applied VirusMuT [6] to estimate the TMRCA by maximum likelihood with a mutation rate of 13.5 per genome per year [7]. The confidence interval was obtained by the bootstrap method. We randomly generated the number of mutations from the Poisson distribution with the parameter of the observed number of mutations and estimated TMRCA using the generated values. The process was repeated 1,000 times to obtain the confidence intervals.

**Estimating the growth rate of the daily confirmed cases at the early stage**

We assume an exponential growth model for the daily confirmed cases at the early stage of the outbreak, and the function is $y=b\exp(kx)$, where *x* denotes days since the initial time of the break, and *y* denotes daily confirmed cases. The loss function was defined as $Loss(k,b)=||y-\hat{y}||_{2}$, where *y* denotes the real daily confirmed cases, and $\hat{y}$ denotes the daily confirmed cases calculated by the exponential growth model.

**Reference**

1. Aksamentov I, Roemer, C., Hodcroft, E. B., & Neher, R. A. Nextclade: clade assignment, mutation calling and quality control for viral genomes. Journal of Open Source Software. 2021;6(67):3773.

2. Kumar S, Stecher G, Li M, Knyaz C, Tamura K. MEGA X: Molecular Evolutionary Genetics Analysis across Computing Platforms. Mol Biol Evol. 2018 Jun 1;35(6):1547-9.

3. Letunic I, Bork P. Interactive Tree Of Life (iTOL) v5: an online tool for phylogenetic tree display and annotation. Nucleic Acids Res. 2021 Jul 2;49(W1):W293-W6.

4. Chi L, Zhang X, Xue Y, Chen H. fastHaN: a fast and scalable program for constructing haplotype network for large-sample sequences. Mol Ecol Resour. 2023 Jun 26. DOI: 10.1111/1755-0998.13829.

5. Murias dos Santos A, Cabezas MP, Tavares AI, Xavier R, Branco M. tcsBU: a tool to extend TCS network layout and visualization. Bioinformatics. 2016 Feb 15;32(4):627-8.

6. Liu Q, Zhao S, Shi CM, Song S, Zhu S, Su Y, et al. Population Genetics of SARS-CoV-2: Disentangling Effects of Sampling Bias and Infection Clusters. Genomics Proteomics Bioinformatics. 2020 Dec;18(6):640-7.

7. Mitja O, Ogoina D, Titanji BK, Galvan C, Muyembe JJ, Marks M, et al. Monkeypox. Lancet. 2023 Jan 7;401(10370):60-74.
