# Supplementary material for: Genomic and epidemiological perspectives on the first local sporadic cases of Mpox in China
Source: Emerg Microbes Infect. 2023 Aug 21;12(2):2245932. doi: 10.1080/22221751.2023.2245932 (PMC10443958; doi:10.1080/22221751.2023.2245932)
Supplement: Supplemental Material [file TEMI_A_2245932_SM3497.docx]

Supplementary Table 1 Characterization and clinical manifestation of the 8 MPXV infected patients in this study

| No. | Gender | Age | Onset Date | Clinical Manifestation | | | | Exposure | | History of STD | MPXV real-time PCR (Ct Value) | | | | Vaccinated against smallpox | MPXV Sequencing | | |
| --- | --- | --- | --- | --- | --- | --- | --- | --- | --- | --- | --- | --- | --- | --- | --- | --- | --- | --- |
|  |  |  |  | Fatigue | Fever (℃) | Rash | Lymphadenectasis | Homosexuality | No. of Mates |  | Rash fluid | Serum | Throat Swab | Environmental Samples |  | Total reads | Mapped reads | Genome coverage % |
| 1 | Male | 43 | 31-May-2023 | Yes | Yes (40.0) | Yes | Yes | Yes | >5 | Syphilis  Genital herpes | + (24.85) | + (39.40) | + (36.92) | NA | Unclear | 34,959,580 | 254,894 | 100.00% |
| 2 | Male | 29 | 29-May-2023 | Yes | No | Yes | Yes | Yes | >5 | Condyloma Acuminatum | + (33.51) | + (38.11) | + (29.37) | NA | No | 60,861,248 | 1,726 | NA |
| 3 | Male | 45 | 3-Jun-2023 | No | No | Yes | Yes | Yes | >3 | Syphilis | + (23.57) | + (37.50) | + (36.42) | + (32.63~39.91) | No | 8,723,587 | 278,438 | 100.00% |
| 4 | Male | 35 | 3-Jun-2023 | No | No | Yes | Yes | Yes | 3 | HIV  Syphilis | + (35.41) | NA | NA | NA | No | NA | NA | NA |
| 5 | Male | 34 | 5-Jun-2023 | No | Yes (38.3) | Yes | Yes | Yes | 1 | HIV  Syphilis  HPV | + (36.72) | NA | NA | - | No | NA | NA | NA |
| 6 | Male | 29 | 4-Jun-2023 | No | No | Yes | Yes | Yes | 1 | HIV  Syphilis | + (38.04) | NA | NA | + (31.69~36.07) | No | NA | NA | NA |
| 7 | Male | 31 | 29-May-2023 | Yes | Yes (38.4) | Yes | Yes | Yes | 1 | HIV | + (38.21) | NA | NA | NA | No | NA | NA | NA |
| 8 | Male | 20 | 6-Jun-2023 | Yes | Yes (38.7) | Yes | Yes | Yes | 2 | No | + (27.99) | NA | NA | + (33.79~36.55) | No | 27,133,714 | 132,822 | 99.97% |

Abbreviations: MPXV, Mpox virus; STD, sexually transmitted disease; NA, not available; HIV, human immunodeficiency virus; HPV, human papilloma virus.

Supplementary Table 2. Information of the three sequences

| Sample ID | Clade | Lineage | # Mutation | # Non-synonymous |
| --- | --- | --- | --- | --- |
| SUB1686660200032 | IIb | B.1.3 | 85 | 37 |
| SUB1686658487091 | IIb | B.1.3 | 86 | 36 |
| SUB1686659883558 | IIb | B.1.3 | 83 | 36 |

Reference Sequence: NC063383.1
